# Ethical and Practical Considerations Associated with Companion Animal Euthanasia

**DOI:** 10.3390/ani13030430

**Published:** 2023-01-27

**Authors:** Kathleen Cooney, Barry Kipperman

**Affiliations:** 1 Companion Animal Euthanasia Training Academy, Loveland, CO 80538, USA; 2 College of Veterinary Medicine, University of Missouri, Columbia, MO 65211, USA

**Keywords:** euthanasia, convenience euthanasia, economic euthanasia, objectionable euthanasia, moral stress, ethical dilemmas, dysthanasia, companion animal, animal hospice

## Abstract

**Simple Summary:**

Companion animal euthanasia is a common procedure in veterinary medicine, intended to end the life of an animal. Veterinarians must determine if the request for euthanasia is warranted or if other factors exist making the decision to euthanize inappropriate or objectionable. If euthanasia is a reasonable course of action, veterinarians must then decide when to euthanize, how to perform the procedure, and other details to protect the mental well-being of all stakeholders. If euthanasia is not elected, veterinarians must also decide how best to support their patient and client.

**Abstract:**

The reality that euthanasia in veterinary practice can end animal suffering but can also be used in circumstances that do not serve an animal’s interest, can be a benefit for animals, and a burden for veterinary professionals, respectively. This essay addresses ethical and practical concerns associated with companion animal euthanasia, including defining euthanasia, why and when euthanasia should be performed, applying euthanasia in practice, contemporary methods, aftercare of deceased animals, and the consequences of euthanasia and dysthanasia for animals, animal owners, and veterinary professionals. We contend that an intention-based definition of euthanasia should be strictly applied in veterinary practice and that practitioners view euthanasia decisions as requests that can (and in some cases should) be declined, rather than as mandates.

## 1. What Is Euthanasia?

The veterinary profession is unique, as its members are expected to end the lives of their patients. To some, this role may conflict with societal perceptions of physicians as healers that should “do no harm” [1]. Veterinarians learn that performing euthanasia is expected during veterinary school and many have experienced it with personal pets. In a study of the cause of death of over 29,000 dogs under primary care in the UK, 89% were euthanized [2]. Euthanasia derives from the Greek roots of “a good death” and in human semantics is restricted to circumstances of mercy killing, in which death is viewed as a respite from inevitable suffering that cannot be alleviated by reasonable means [3]. The expectation is that if life is to be taken, it is at the right time for the right reason.

The American Veterinary Medical Association (AVMA) guidelines for the euthanasia of animals define the following co-dependent, necessary requirements for euthanasia:

1. Method-based: “the use of humane techniques to induce the most rapid and painless and distress-free death possible”;

2. Intention-based: “to induce death in a manner that is in accord with an animal’s interest and/or because it is a matter of welfare” [4].

However, the guidelines address the intentions associated with euthanasia equivocally, and thereby diminish the significance of this justification for the act, noting, “The Panel believes that evaluating... the rationale for inducing death… is beyond its purview” [4]. Even if an animal’s “best interest” paradigm is used to justify euthanasia, Quain notes difficulties in applying this principle: “Different stakeholders (for example, the veterinarian and the owner of the animal, the veterinarian and colleagues, or two different owners of the same animal) may have different views about an animal’s quality of life, prognosis and interests” [5].

Rollin [6] has observed that euthanasia is a double-edged sword, referring to its benefit in ending terminal suffering, but it can also be performed in circumstances that appear less compulsory and do not conform with serving an animal’s best interest. Tannenbaum refers to euthanasia that does not align with an animal’s best interest as “medically unnecessary euthanasia” [7]. Convenience euthanasia refers to requests to end the life of an animal due to unexpected changes in the owner’s life, insufficient time, capability (emotional and physical), or motivation to care for an animal. [8,9]. Others may object to this term on the premise that the owner may not disclose all the concerns that contributed to this decision and that the practitioner has a duty to abide by the wishes of the client paying for their services. Most veterinarians consider euthanasia of a healthy animal to conflict with their role as patient advocate [10]. With the potential for significant caregiver burden brought on by a pet’s condition, veterinarians are regularly tasked with deciding whose suffering is worse, the client’s or the pet’s.

From 1998 to 2014, veterinary prices in the US rose faster than the rate of inflation [11] and in the past year fees rose 10%, the largest spike in two decades [12]. In a study of US small animal veterinarians, 57% reported that economic limitations of owners adversely affected their ability to provide the quality of care they would like at least once or multiple times per day [13]. Economic euthanasia is defined as a circumstance in which “euthanasia is elected based primarily, …or to a large degree on the cost of veterinary …care; or a condition in which veterinary care is sought and minimal or no testing/treatment is elected based on the costs of care, resulting in eventual euthanasia” [14].

Quain has defined ethically indicated euthanasia as “euthanasia performed in what are believed to be the animal’s interests and which is not considered to be primarily motivated by convenience, economics or reasons to which veterinary team members object” [5]. We suggest that euthanasia that does not meet this standard be referred to as convenience, economic, or objectionable euthanasia. Utilization of the term euthanasia (without a preceding qualification) in settings in which veterinarians are asked to cause the intentional death of healthy companion animals that are unwanted or of sick animals that could be restored to health by routine veterinary interventions obfuscates its meaning and should be discouraged.

Yeates warns of the potential consequences of the inappropriate use of the term euthanasia:

*Using the term ‘euthanasia’ might make us feel better about doing it. But it might also hide some moral concerns, insofar as ‘euthanasia’ is a label that implies morally acceptable (or even laudable) killing. In cases that stretch or miss that definition, this might mislead people, and diminish the inclination to consider the moral aspects of the decision, making the thus labelled killing more prevalent.*

[15]

## 2. Why Euthanasia Should Be Performed

Tannenbaum cites the following four criteria to justify euthanasia:

*(1) the patient is suffering from a condition for which veterinary medicine cannot offer a cure or solution at any cost; (2) the condition is already causing the animal severe pain for which palliative measures… are not available; (3) the client is …able to make a voluntary and rational decision; (4) the client… [understands] that the animal’s interest in freedom from suffering ought to take precedence over… [their] own impending grief or sense of loss.*

[7]

There will be many instances when a veterinarian must determine if euthanasia is the best course of action (or is the most reasonable course) based on limited information, such as a physical examination alone, without the benefit of a diagnosis or response to treatment. Veterinarians are frequently expected to predict the future outcomes of their patient’s conditions or even when a patient’s natural death would be expected to occur. These uncertainties often accompany a burden of doubt for veterinarians regarding if euthanasia should be elected. While there are many contributing factors for why euthanasia is chosen (immobility, behavior changes, lethargy, etc.), there are few objective tools to determine exactly when euthanasia is needed. Presently, the decision to euthanize is based on a veterinarian’s assessment of the facts presented to them. The decision may be further shaped by their previous experiences, beliefs, and biases.

Veterinarians tend to be familiar with quality of life (QOL) scales that seek to objectively measure when euthanasia is warranted to avoid negative states of suffering, such as pain, physiologic and emotional distress, and anxiety [16]. Unfortunately, they are subjective to the point that one household may score their pet’s QOL very differently than another household in a similar situation. Veterinarians may use QOL scales to open dialogue with owners about disease trajectories but must still rely on their instinct and medical acumen to guide what’s best for their patient.

Euthanasia should be performed when no other options exist to address significant and permanent suffering. Not every manifestation of suffering warrants euthanasia. It will depend on the degree of distress the animal experiences and how it copes. A cat or small dog unable to walk may still have a satisfactory QOL with the help of its family while a recumbent dog with pressure sores and decubital ulcers may not. Owners may define suffering based on psychosocial factors, such as previous experiences and beliefs. Their determination if their pet is suffering is as relevant (if not more so) as that of the veterinarian. The difference rests in the veterinarian’s knowledge of diseases, the impacts on animal welfare, and available treatments. If agreement cannot be reached, an animal may suffer or die prematurely through euthanasia. Animal hospice and the use of palliative medicine should be considered any time suffering is expected before euthanasia is chosen.

## 3. Application of Euthanasia in Practice

It is to be expected that a veterinary practitioner will encounter conflicts about the suitability of euthanasia requests. In a survey of North American veterinarians, 93% received what they considered to be an inappropriate request for euthanasia [17]. In a study of small animal veterinarians, euthanasia requests perceived to be based on lack of financial means occurred with a median frequency of once a month [18]. In the same report, 45% of veterinarians agreed that veterinarians sometimes use euthanasia as an aid or method to resolve difficult cases when this may not be in the best interest of the patient, and 42% of practitioners reported that they had done this at least once in their career. These results confirm that companion animal clinicians perform euthanasia when this is not warranted, based on animal welfare considerations. This reflects the reality that veterinarians have obligations to the interests of animals, their owners, and themselves, and these often conflict [19].

Complicating euthanasia decisions is the fact that companion animals are legal property, and barring violations of animal cruelty laws, are subject to the owner’s disposition. Autonomy is considered a fundamental bioethical principle [20] but whose autonomy should be prioritized when considering euthanasia? A veterinarian may choose to comply with euthanasia requests, dissuade the animal’s owner, or decline such requests. In a study of UK veterinarians, 71% reported wanting to refuse a request but performed euthanasia anyway [21]. Pressure from a superior was given as a reason for proceeding with euthanasia. Veterinarians in the US commented that they felt discouraged from exercising their discretion to refuse euthanasia requests and sensed significant peer pressure from employers or hospital administrators to comply with euthanasia requests that conflicted with their personal values [22].

In a study of small animal veterinarians, 80% reported to have declined a euthanasia request at some point in their career: of these, 52% reported that they decline euthanasia requests every few years [18]. A survey of UK veterinarians found that 81% had refused to perform euthanasia, with the median frequency of refusal “yearly or less” [21]. There is no data to indicate animal welfare outcomes of these cases. The relative infrequency with which practitioners decline euthanasia requests conflicts with the AVMA guidelines: “There may be instances in which the decision to kill an animal is questionable, especially if the animal is predicted to have a life worth living. In this case, the veterinarian, as …animal advocate, should be able to speak frankly about the animal’s condition and suggest alternatives to euthanasia” [4]. Asking owners “What would we do if euthanasia wasn’t an option?” might be of use to veterinarians. This could open dialogue about reasonable alternatives that better meet the animal’s needs while reducing moral stress for veterinarians, knowing they advocated for what they felt was in their patient’s best interest.

The most common reasons acknowledged for why veterinarians are hesitant to decline euthanasia requests included concerns that the owner may pursue other alternatives which might worsen the welfare of the animal, their difficulty declining such requests once an owner had reached this decision and concern that declining euthanasia requests may jeopardize their relationship with the owner [18]. Interestingly, veterinarians who reported prioritizing patient interests relative to client interests were significantly more likely to decline euthanasia requests. We contend that avoiding difficult decisions or retaining a client are not worth the moral stress that may occur as a consequence of performing convenience, economic, or objectionable euthanasia.

Tannenbaum implores veterinarians to view euthanasia decisions as arising from requests rather than mandates:

*Getting into the habit of agreeing to clients’ requests for euthanasia can put one in a frame of mind in which alternatives might not be explored vigorously. A profession allowed by law, its own official ethical standards, and societal attitudes to kill its patients may well kill too many patients. It is not in the …interests of the profession to perpetuate an image of itself as willing to kill any companion animal on demand. Veterinarians who kill on demand are sending a message to clients and the public that their patients are not worth much.*

[7]

The reality that euthanasia in veterinary medicine can end animal suffering but can also be used in circumstances that do not serve an animal’s interest, can be a benefit for animals, and a burden for veterinary professionals, respectively. Acting in ways that one believes to be suspect or wrong (i.e., euthanasia), based on the belief that another person (the animal’s owner) may do something that leads to a poorer outcome for the animal, is an inadequate supposition to base ethical decisions on. We are not responsible for the choices and actions of others. Veterinarians must instead be accountable for the consequences of their behaviors. However, because veterinarians have compassion for both animals and their owners, it is very difficult to tell an owner “I’m not comfortable complying with your request”. Ethical behavior requires courage. We propose that all practices should have a policy or standard operating procedure supporting declination of euthanasia requests on moral grounds.

The AVMA guidelines are ambiguous regarding the basis of euthanasia decisions, noting, “Impacts on animals may not always be the center of the valuation process, and there is disagreement on how to account for conflicting interspecific interests” [4]. An intention-based definition of euthanasia that centers on benefiting the recipient may better ensure that such decisions would be based primarily from the perspective of the animal and may serve to reduce the prevalence of decisions to end a companion animal’s life as a means of resolving dilemmas between serving the owner’s, patient’s, and the veterinarian’s interests. An intention-based definition of euthanasia may provide clearer guidance to veterinarians as they confront euthanasia decisions.

## 4. When Euthanasia Should Be Performed

Even if one can confidently justify on moral grounds why an animal should be euthanized, it can be very difficult to know when euthanasia should be performed. One factor relevant to the timing of euthanasia is the question of whether veterinarians should initiate this difficult topic or wait for owners to do so. We believe it is the responsibility of the compassionate veterinarian to raise the topic of euthanasia when indicated and that abdication of this duty is likely to prolong animal suffering.

Because the need for euthanasia may arise during any life stage, it is reasonable to open dialogue about end-of-life options before euthanasia is necessary, especially for patients with congenital diseases, relatively short life spans, and when chronic disease is diagnosed. While broaching the subject of death and dying is challenging [23], veterinarians uncomfortable with such discussions are missing an important opportunity to educate owners on what to expect and what to prepare for as their pet reaches the end of life. Pre-planning details around euthanasia are ranked among the top five components of a good death experience according to owners [24]. Veterinary services have yet to tap into the potential for education on various aspects relevant to end-of-life [25]. This is a disservice for owners seeking to learn about euthanasia, hospice, and aftercare. Providing owners with patient symptoms to guide when palliative medicine is necessary or criteria for when euthanasia is justified (i.e., not eating any food, vomiting daily) can be very helpful as well.

Owners concerned about imminent crisis events may prefer to euthanize an animal sooner rather than later to spare themselves the distress of watching their companion decline and the emotions that occur from fear of the unknown. From a welfare perspective, advocating for euthanasia before a crisis event occurs is beneficial to the animal who avoids harmful states, such as breathing difficulty or severe pain. However, choosing euthanasia based on the assumption of a crisis is flawed. Veterinarians have an ethical responsibility to educate owners about disease symptoms and trajectories for all patients, including the likelihood and nature of possible crisis events, and whether and how these can be addressed, including costs.

It is common after discussion of euthanasia for owners to elect to take the animal home to allow them time to accept the situation, and sometimes, for other family members to say goodbye. In some cases, the timeline proposed by the animal’s owner may be measured in weeks when the veterinarian believes that a more appropriate timetable for euthanasia should be measured in hours or days. This circumstance creates ethical challenges for the veterinarian who wishes to advocate for the interests of the suffering patient who will benefit from death, yet also respect the difficulty for the owner letting go of a beloved family member. We believe that veterinarians must summon the courage to advocate for the timely euthanasia of suffering patients, even when this conflicts with owner desires. None of us want to part with a beloved animal companion. Animal owners rely on veterinarians as a source of objectivity and to be the animal’s advocate to guide this difficult decision.

Animal hospice can alleviate suffering in many cases through the application of palliative medicine, and by the ability of the owner to afford it. If veterinarians are honest and forthcoming about projections around death and dying, owners can feel more empowered to make sensible decisions for their pet, themselves, and their family. This equates to a more controlled decision about timing of euthanasia for all stakeholders. A strong comprehension of hospice and palliative care, and how to apply it in practice, reduces the likelihood of owner coercion to elect euthanasia.

What about the circumstance in which a veterinarian sees an animal who is clearly suffering and would benefit from hospice care or euthanasia, whose owner declines these options? While AVMA guidelines [26] clearly specify the responsibility of veterinarians to report suspected animal cruelty, and while reporting is mandated in some states [27], practitioners are typically reluctant to do so [28] and tend to reserve reporting for cases of cruelty or neglect unrelated to advanced medical conditions. In our experience, clinicians almost never report an owner whose animal is in terminal condition who wishes for a natural death to occur at home or who simply cannot decide what to do.

Lastly, there may be instances when euthanasia is performed due to veterinarian availability. For example, we see this situation arise before the close of a shift, before a vacation, or perhaps when a preferred home euthanasia can be performed. Fear can be the primary factor; fear of what would happen to the animal if the veterinarian wasn’t available to euthanize, and in the manner desired by the owner. We challenge veterinarians to seek alternative solutions to euthanasia based on veterinarian convenience, such as delegating to an associate, leveraging local community veterinary services, and increasing use of hospice and palliative care until such time as euthanasia should be provided.

## 5. How to Perform Euthanasia

Euthanasia can be carried out by several methods, all of which aim to cease life in a swift and efficient manner with the least amount of hardship for the animal. For companion animals, the most common method is via chemicals/drugs (e.g., pentobarbital sodium). Euthanasia methodology with attention to patient comfort has advanced significantly in the past decade with the intention of providing a better experience for animals, owners, veterinarians, and involved veterinary personnel. Past approaches emphasized the rapid and efficient administration of euthanasia solution in conscious, alert animals; while effective, animal welfare concerns included anxiety and pain from placement of an intravenous (IV) catheter and/or from the injection itself, such as inadvertent extravasation. The AVMA Euthanasia guidelines state that euthanasia should avoid pain and distress [4]. Recently, the paradigm has shifted from the primary goal being a fast death, to a more calm and controlled death with loved ones present.

Bioethicist Jessica Pierce advocates for adding a sixth freedom to the five freedoms of animal welfare [29]: the freedom to die a good death. “A good death is one that is free of unnecessary pain, suffering, and fear; it is peaceful; and it takes place in the presence of compassionate witnesses” [30]. When euthanasia is elected, veterinarians have an ethical responsibility to provide all patients a good death, especially because there is only one opportunity to get it right. Owners want the option to be with their pet for the entirety of the appointment (never separated), to be provided with details to help them prepare, to see their pet sleeping with little to no experienced pain or anxiety throughout the procedure, and to be given the option of home euthanasia whenever possible [24]. The evolving status of animals as family members means that companion animal euthanasia is now more of an experience with multiple components rather than a singular medical act.

A good death includes proper delivery of the medication. The administration of a euthanasia solution to achieve irreversible brain death and cardiac arrest has predominantly been carried out via an IV injection. In US veterinary schools, this remains the primary technique taught to veterinary students [31]. However, other techniques exist [32] and it is a veterinarian’s duty to understand what they are, their indications (such as when insertion of an IV catheter is untenable), and how to perform them. Knowing which technique to attempt based on patient signalment and supplies, reduces complications and increases the likelihood of success the first time. It is also important to consider these techniques in order of what provides the least pain/anxiety, what will be most reliable/irreversible, and what is safest for aftercare management, e.g., presence of pentobarbital in tissues.

Avoiding a bad death (i.e., dysthanasia) becomes as important as performing euthanasia well. Consequently, preventing errors during the euthanasia procedure should be a foremost consideration of the veterinarian. The use of standard operating procedures, (SOPs) based on peer-reviewed clinical practice guidelines, helps build consistency in the delivery of care. The 2016 IAAHPC/AAHA end-of-life care guidelines offer clear instruction on how to perform euthanasia in both the hospital and home setting [33]. Many veterinarians rely on SOPs to help ensure consistency and quality of their services, and many hospitals are accustomed to their use for activities such as surgery and practice management [34]. Errors can also be prevented with greater attention to medical record keeping both before and after euthanasia, such as documentation of conversations leading up to euthanasia, what drugs were used for the procedure, and the manner of the animal’s death [35,36].

Veterinary appointments often range from 15–20 min [37]. Such short times limit the application of modern euthanasia methods, which include what the Companion Animal Euthanasia Training Academy (CAETA) refers to as the 14 essential components of a good death (Table 1) [38]. The components are a compilation of recommendations from various sources intended to benefit patients, owners, and the veterinary team. These include time to establish rapport and trust, outlining caregiver and pet preferences for a smoother experience, use of pre-euthanasia sedatives/anesthetics/anxiolytics, privacy, and inclusion of loved ones.

Rushing through key components has left owners frustrated and upset [24]. Companion animal euthanasias have evolved to be pseudo-funerals; unique, emotional medical procedures in full view of owners unlike anything else undertaken in veterinary medicine. Lengthening scheduled euthanasia appointments as the new normal is warranted to accommodate all the necessary components of modern euthanasia. We propose that longer appointment times for all sick patients in the range of 45–60 min become the standard, as some of these appointments may result in a euthanasia decision: if not, this still improves the veterinarian’s capacity to provide informed consent, discuss prognosis, and achieve shared decisions [39].

## 6. Ethical Pet Aftercare

It is common for veterinary teams to make deceased pet aftercare arrangements on behalf of their client. When an animal dies, the body is typically managed by the veterinary team, who sets into motion a series of owner requests, most commonly for cremation [40]. Cremation is the process of reducing a body to ash through burning, which occurs at a crematory or crematorium. Necessary details include cremation type, urn selection, memorialization item, and ash return location. The veterinary hospital orders these requests and is considered by the aftercare company to be their client, not the animal owner.

This leads to important ethical considerations. One is transparency in choices made by veterinarians and how they select aftercare arrangements. Many veterinary hospitals have contracts with a singular crematory company with an established pricing structure for cremation services. Such relationships are often based on precedent rather than an evaluation of comparative quality or reputation. While useful in consistency for staff and record keeping, this may discourage veterinarians from working with another facility. This raises concerns when an owner has already selected a crematory with whom the veterinarian is not familiar. The veterinarian may steer the client towards the one they prefer working with. Even larger ethical concerns are raised if the veterinarian receives higher financial gain when working with one crematory over another. Veterinarians should also describe the type of cremation (ex. private, semi-private, communal), or alternatives to cremation such as aquamation, with accuracy so owners are not accidentally or intentionally misled [41].

How the body is handled while in the care of the veterinarian also deserves attention. Clients have voiced their preference for body handling/storage, choosing more respectful containers (e.g., specially designed transport bags) over the commonly used plastic refuse bags [40], yet these bags are still regularly used in practice. Pet crematories report that many veterinarians routinely bring their own pets directly to the crematory to avoid placing their pet in the hospital cooler and to avoid placing them in refuse bags. Hospitals typically use freezers to hold client-owned animals until the crematory service can pick them up, a less-than-ideal system for those who would prefer the body arrive expeditiously to its destination. In our experience, veterinarians shield owners from witnessing how the deceased’s body is contained and stored until crematory pickup, perhaps due to their own disapproval of current norms. When we know better, we do better, and veterinarians have an opportunity to elevate ethical behaviors related to the aftercare of deceased patients, treating the body as if it were their own pet.

Dead bodies can be considered to have intrinsic and instrumental value [42]. They represent an individual who lived but can also be used to advance the education of others. Globally, deceased humans have been used to teach students and physicians for centuries. In the past, unethical sources included acquisition from the poor and from unclaimed individuals [43]. A bioethical paradigm is that it is disrespectful to do something to a person’s body without their consent or that of a family member.

A comparable and transparent ethos should apply to deceased animals. Use of deceased animals for teaching and training purposes should be limited to those in which informed consent is acquired from the owner. It may once have been considered acceptable to routinely practice learning exercises on cadavers without their owner’s awareness to advance medical knowledge, but professional ethics prompts reconsideration of this behavior. In our experience, owners have been receptive to donating their pet’s body for select teaching purposes and have signed agreements indicating such consent. For all approaches to aftercare, veterinarians should ask themselves, “Would I be willing to inform my clients or my colleagues of these practices?”

## 7. Consequences of Companion Animal Euthanasia

Performing euthanasia can engender both positive and negative emotional experiences. The negative effects have been widely explored because of their potential to have detrimental impacts on the mental health of veterinarians and support staff. Multiple studies have concluded that performing euthanasia can have harmful psychological consequences for veterinarians, including burnout, compassion fatigue, and reduced career satisfaction [44,45]. People who euthanize animals can develop perpetration-induced traumatic stress, a type of post-traumatic stress disorder [46,47]. Indirect exposure to euthanasia-associated stress, i.e., secondary traumatic stress, is also experienced by veterinary support staff associated with witnessing the physical and/or emotional trauma of the patient and/or owner [48]. As the entire veterinary team is therefore vulnerable to emotional harm associated with euthanasia, management needs to broaden monitoring beyond just those performing the procedure. Further research of the experiences of veterinary support teams related to euthanasia is warranted.

A frequent outcome of euthanasia for veterinarians is moral [dis]stress [17,18]. Moral distress is, “The experience of psychological distress that results from engaging in, or failing to prevent, decisions or behaviors that transgress, …personally held moral or ethical beliefs” [49]. Moral stress is a potential result of experiencing conflicts associated with work-related duties or expectations that do not align with one’s values [50].

Rollin [51] first established the concept of moral stress among veterinary professionals related to killing animals for reasons deemed to be objectionable: “The stress … of killing healthy animals (or being asked to kill them even if one refuses to do so) is, in my experience, the most demoralizing part of [veterinary] practice. It arises out of a fundamental conflict between one’s reasons for going into animal work and what one is in fact doing or being asked to do”.

Contributing factors to moral stress associated with killing animals who could maintain a good quality of life include a deep respect for animals by veterinary professionals, the realization that death is not in accord with the animal’s interest, the belief that one must repress their moral indignation at those who created the ethical conflict, and a reluctance to discuss the issue with friends or family [6]. Morris summarizes this quandary: “Veterinary work often causes distress …because it requires people who care strongly for animals to kill them when they are not sick enough to easily justify their death” [52].

In one report, 68% of small animal veterinarians had regrets about a euthanasia decision [22]. In a recent study of veterinarians, 84% experienced sadness associated with euthanasia, 70% felt frustration, 67% had doubts about the procedure, and 59% felt guilt [53]. Another study found that 40% of veterinarians reported that their mental and physical health had been affected by euthanasia and 34% had trouble performing euthanasia because of personal distress [54].

Given the relationship between euthanasia requests that practitioners may find unethical and moral stress, it is quite understandable for veterinary professionals to develop emotionally protective strategies to ease this burden. Bartram and Baldwin proposed that veterinarians experience unpleasant cognitive dissonance between their desire to heal patients and their incapacity to successfully treat patients [55], which could be mitigated by modifying their values to see euthanasia as a favorable conclusion. Consequently, veterinarians may develop detachment from the event and rationalize that euthanasia is the best alternative for the animal to cope with difficult end-of-life decisions [52].

A possible consequence of these adaptations is that veterinarians may change their attitudes toward euthanasia, coming to perceive it as more acceptable. A UK study confirms this, finding that pre-clinical veterinary students were less tolerant of convenience euthanasia, compared to veterinarians and veterinary students in the later stages of training [56]. In another study, Austrian veterinarians with more experience were more likely to agree with convenience euthanasia [57]. Veterinarians over time may become desensitized to the profound implications of euthanasia, seeing it as one of many routine, socially sanctioned options to provide owners of sick animals.

There has been increasing concern regarding an elevated risk of suicide among veterinarians, compared with comparable occupations [58]. Two studies did not support an association between performing euthanasia (including objectionable euthanasia) and suicide risk [59,60]. Suicide death rates among veterinarians have been linked to access to controlled substances, in particular the euthanasia drug pentobarbital, and the knowledge of how to use them [61,62]. It has been proposed [62] that creating safeguards to controlled substance access (such as ensuring that two different staff members sign off) may reduce the likelihood of suicide attempts and death, without increasing barriers to clinical work. Do veterinary practice owners and hospital administrators have an ethical responsibility to implement steps to protect vulnerable veterinary personnel? This certainly seems to be a reasonable precautionary measure and should become commonplace within veterinary practices.

Compassion satisfaction is the opposite of compassion fatigue. It is the pleasure and positive feelings that come from helping others and from finding meaning and fulfillment in work [63]. In veterinary medicine, this includes euthanasia-centric services, when euthanasia is deemed beneficial to the patient and moral stress is minimal. Medically indicated euthanasia is often viewed as the final act of love an owner can provide for their animal companion. Quain has referred to this as “the gift” of ending suffering [5]. Many practitioners observe that they receive more cards and letters from owners expressing appreciation related to euthanasia than for all other possible reasons.

To only focus on the effects of euthanasia on the mental health of veterinary professionals would be a disservice to animal owners. They expect a death process for their companion free of pain and fear and define ‘a good death’ beyond just the procedure itself [24]. When an animal is considered a member of the family, making the decision to end its life can be a very distressing and protracted process, often described by owners as the hardest thing they have ever had to do. Owners reasonably expect emotional support during this time, including validation of their decision (if this can be offered sincerely) which mitigates feelings of guilt [64].

Owners can become emotionally traumatized by negative experiences associated with euthanasia or ‘dysthanasia’ (such as atypical vocalizations, screams, twitching, patient tremors, and feeling rushed) and have shared these concerns with CAETA. As a result, CAETA has developed a Euthanasia Review Department to field questions and concerns raised by owners and veterinarians. A ‘dysthanasia’ may cause a reluctance by owners to elect euthanasia of other animals in the future and create a negative perception of the veterinary profession as uncaring or incompetent.

## 8. Conclusions

Companion animal euthanasia is fraught with emotions and ethical challenges which can lead to moral and traumatic stress for veterinary professionals and animal owners, respectively. We define euthanasia as the intentional ending of life primarily to benefit the animal’s interest only when suffering is imminent or permanent and cannot be alleviated. We should strive for alternatives, such as cures for diseases or palliative medicine to delay euthanasia for as long as possible. On a positive note, paying more attention to euthanasia training and education has far reaching benefits for all stakeholders. There is much to be learned from seasoned experts who have devoted their careers to practicing euthanasia and from ethicists, who can guide practitioners on best practices through a solid ethical approach, respectively. While making the decision to euthanize an animal companion may be difficult due to limited information and uncertainty about a patient’s condition, veterinarians should ensure a peaceful death, establish an ethical approach to aftercare, and demonstrate compassion to grieving animal owners, the veterinary team, and themselves.

## Figures and Tables

**Table 1:** The 14 essential components of a companion animal euthanasia (CAETA) [38].

G	Grief support materials provided
O	Outline caregiver and pet preferences
O	Offer privacy before and after death
D	Deliver proper technique
E	Establish rapport
U	Use of pre-euthanasia sedation or anesthesia
T	Thorough, complete consent
H	Helpful and compassionate personnel
A	Adequate time
N	Narrate the process
A	Avoid pain and anxiety
S	Safe space to gather
I	Inclusion of loved ones
A	Assistance with body care

## Data Availability

Not applicable.

## References

[1] Deguma J.J., Deguma M.C., Añora H.C., Loremia V.Z., Cabigon A.F.P., Case H.S. (2020). Why is it better to suffer and not to die through euthanasia? A multi-perspective analysis. J. Soc. Political Sci..

[2] Pegram C., Gray C., Packer R., Richards Y., Church D.B., Brodbelt D.C., O’Neill D.G. (2021). Proportion and risk factors for death by euthanasia in dogs in the UK. Sci. Rep..

[3] Dyck A., Kohl M. (1975). Beneficent Euthanasia and Benemortasia: Alternative Views of Mercy. Beneficent Euthanasia.

[4] Leary S., Underwood W., Anthony R., Cartner S., Grandin T., Greenacre C., Gwaltney-Brant S., McCrackin M.A., Meyer R., Miller D. (2020). AVMA Guidelines for the Euthanasia of Animals, 2020 ed.

[5] Quain A. (2021). The Gift: Ethically indicated euthanasia in companion animal practice. Vet. Sci..

[6] Rollin B.E. (2011). Euthanasia, moral stress and chronic illness in veterinary medicine. Vet. Clin. Sm. Anim..

[7] Tannenbaum J. (1995). Veterinary Ethics: Animal Welfare, Client Relations, Competition and Collegiality.

[8] Batchelor C.E.M., McKeegan D.E. (2012). Survey of the frequency and perceived stressfulness of ethical dilemmas encountered in UK veterinary practice. Vet. Rec..

[9] Rathwell-Deault D., Godard B., Frank D., Doize B. (2017). Conceptualization of convenience euthanasia as an ethical dilemma for veterinarians in Quebec. Can. Vet. J..

[10] Rollin B.E. (2006). An Introduction to Veterinary Medical Ethics.

[11] Brake R., Dicks M. The Pricing Struggle: How High Is Too High for Pet Owners? DVM360 Magazine 2017. https://www.avma.org/sites/default/files/resources/2017-Aug-3_The-pricing-struggle_How-high-is-too-high-for-pet-owners_.pdf.

[12] Rugaber C. (2022). What Spiking US Veterinary Prices Reveal about Inflation. Associated Press. https://www.usnews.com/news/business/articles/2022-10-12/what-spiking-us-veterinary-prices-reveal-about-inflation.

[13] Kipperman B.S., Kass P.H., Rishniw M. (2017). Factors that influence small animal veterinarians’ opinions and actions regarding cost of care and effects of economic limitations on patient care and outcome and professional career satisfaction and burnout. J. Am. Vet. Med. Assoc..

[14] Kipperman B. (2010). Economic Euthanasia: A Disease in Need of Prevention. https://www.hsvma.org/economic_euthanasia_disease_in_need_of_prevention#.Y1mdNC-B30o.

[15] Yeates J.W., Kipperman B., Rollin B.E. (2022). Death. Ethics in Veterinary Practice.

[16] Villalobos A.E. (2011). Quality-of-life Assessment Techniques for Veterinarians. Vet. Clin. North America Small Anim. Pract..

[17] Moses L., Malowney M.J., Boyd J.W. (2018). Ethical conflict and moral distress in veterinary practice: A survey of North American veterinarians. J. Vet. Int. Med..

[18] Kipperman B., Morris P., Rollin B. (2018). Ethical dilemmas encountered by small animal veterinarians: Characterization, responses, consequences and beliefs regarding euthanasia. Vet. Rec..

[19] Kipperman B., Kipperman B., Rollin B.E. (2022). Veterinary advocacies and ethical dilemmas. Ethics in Veterinary Practice.

[20] Beauchamp T.L., Childress J.F. (2019). Principles of Biomedical Ethics.

[21] Yeates J.W., Main D.C.J. (2011). Veterinary opinions on refusing euthanasia: Justifications and philosophical frameworks. Vet. Rec..

[22] Kipperman B. (2017). Ethical Dilemmas Encountered by Small Animal Veterinarians: Characterization, Responses, Consequences and Beliefs Regarding Euthanasia. Master’s Thesis.

[23] Shaw J.R., Lagoni L. (2007). End-of-Life Communication in Veterinary Medicine: Delivering Bad News and Euthanasia Decision Making. Vet. Clin. North America Small Anim. Pract..

[24] Cooney K., Lori K. (2022). How Pet Owners Define a ‘Good Death’: New Study Reveals Some Surprising Facts. DVM360 Mag..

[25] Kogan L. (2021). Mention the Unmentionables. Today’s Veterinary Business. https://todaysveterinarybusiness.com/end-of-life-veterinary-care/.

[26] American Veterinary Medical Association Animal Abuse and Animal Neglect. https://www.avma.org/resources-tools/avma-policies/animal-abuse-and-animal-neglect.

[27] Wisch R.F. (2020). Table of Veterinary Reporting Requirement and Immunity Laws. Animal Legal & Historical Center, Michigan State University. https://www.animallaw.info/topic/table-veterinary-reporting-requirement-and-immunity-laws.

[28] Kogan L.R., Schoenfeld-Tacher R.M., Hellyer P.W., Rishniw M., Ruch-Gallie R.A. (2017). Survey of attitudes toward and experiences with animal abuse encounters in a convenience sample of US veterinarians. J. Am. Vet. Med. Assoc..

[29] FAWC (Farm Animal Welfare Council) 2012 Archived. Five Freedoms. https://webarchive.nationalarchives.gov.uk/20121010012427/www.fawc.org.uk/freedoms.htm.

[30] Pierce J. (2012). The Last Walk: Reflections on Our Pets at the End of Their Lives.

[31] Dickinson G., Hoffmann H., Cooney K. (2021). Euthanasia Education in Veterinary Schools in the United States. J. Vet. Med. Educ..

[32] Cooney K. (2020). Common and Alternative Routes of Euthanasia Solution Administration. Vet. Clin. North America Small Anim. Pract..

[33] Bishop G., Cooney K., Cox S., Downing R., Mitchener K., Shanan A., Soares N., Stevens B., Wynn T. (2016). 2016 AAHA/IAAHPC End-of-Life Care Guidelines. J. Am. Anim. Hosp. Assoc..

[34] Freeman K.P., Cook J.T., Hooijberg E.H. (2021). Standard Operating Procedures. J. Am. Vet. Med. Assoc..

[35] Gray C., Radford A. (2022). Using Electronic Health Records to Explore Negotiations Around Euthanasia Decision Making for Dogs and Cats in the UK. Vet. Rec..

[36] Cooney K.A. (2022). Importance of Documenting Euthanasia Decision-making Processes in Patients’ Medical Records. Vet. Rec..

[37] Corah L., Lambert A., Cobb K., Mossop L. (2019). Appointment Scheduling and Cost in First Opinion Small Animal Practice. Heliyon.

[38] Companion Animal Euthanasia Training Academy (2019). What’s Behind a Good Pet Euthanasia?. https://caetainternational.com/whats-behind-a-good-pet-euthanasia/.

[39] Janke N., Coe J.B., Bernardo T.M., Dewey C.E., Stone E.A. (2021). Pet owners’ and veterinarians’ perceptions of information exchange and clinical decision-making in companion animal practice. PLoS ONE.

[40] Cooney K., Kogan L., Brooks S., Ellis C. (2021). Pet Owners’ Expectations for Pet End-of-Life Support and After-Death Body Care: Exploration and Practical Applications. Top. Companion Anim. Med..

[41] Ellis C. (2017). Aftercare. Hospice and Palliative Care for Companion Animals.

[42] Champney T.H., Hildebrandt S., Gareth Jones D., Winkelmann A. (2019). Bodies R us: Ethical views on the Commercialization of the dead in medical education and research. Anat. Sci. Educ..

[43] Champney T.H. (2019). A bioethos for bodies: Respecting a priceless resource. Anat. Sci. Educ..

[44] Scotney R.L., McLaughlin D., Keates H.L. (2015). A systematic review of the effects of euthanasia and occupational stress in personnel working with animals in animal shelters, veterinary clinics, and biomedical research facilities. J. Am. Vet. Med. Assoc..

[45] Jacobs J., Reese L.A. (2021). Compassion fatigue among animal shelter volunteers: Examining personal and organizational risk factors. Anthrozoös.

[46] MacNair R. (2002). Perpetration-Induced Traumatic Stress: The Psychological Consequences of Killing.

[47] Bennett P., Rohlf V. (2005). Perpetration-induced traumatic stress in persons who euthanize nonhuman animals in surgeries, animal shelters, and laboratories. Soc. Anim..

[48] Hanrahan C., Sabo B.M., Robb P. (2018). Secondary Traumatic Stress and Veterinarians: Human-Animal Bonds as Psychosocial Determinants of Health. Traumatology.

[49] Crane M.F., Bayl-Smith P., Cartmill J. (2013). A recommendation for expanding the definition of moral distress experienced in the workplace. Aust. NZ J. Organ. Psych..

[50] Fawcett A., Mullan S. (2018). Managing moral distress in practice. In Practice.

[51] Rollin B.E. (1987). Euthanasia and moral stress. Loss Grief Care.

[52] Morris P. (2012). Blue Juice: Euthanasia in Veterinary Medicine.

[53] Deponti P.S., Jaguezeski A.M., Pulgatti D.H.V., Soares J.C.M., Cecim M.D.S. (2023). Veterinarian’s perceptions of animal euthanasia and the relation to their own mental health. Ciência Rural..

[54] Dow M.Q., Chur-Hansen A., Hamood W., Edwards S. (2019). Impact of dealing with bereaved clients on the psychological wellbeing of veterinarians. Aust. Vet. J..

[55] Bartram D.J., Baldwin D.S. (2010). Veterinary surgeons and suicide: A structured review of possible influences on increased risk. Vet. Rec..

[56] Ogden U., Kinnison T., May S.A. (2012). Attitudes to animal euthanasia do not correlate with acceptance of human euthanasia or suicide. Vet. Rec..

[57] Hartnack S., Springer S., Pittavino M., Grimm H. (2016). Attitudes of Austrian veterinarians towards euthanasia in small animal practice: Impacts of age and gender on views on euthanasia. BMC Vet. Res..

[58] Tomasi S.E., Fechter-Leggett E.D., Edwards N.T., Reddish A.D., Crosby A.E., Nett R.J. (2019). Suicide among veterinarians in the United States from 1979 through 2015. J. Am. Vet. Med. Assoc..

[59] Glaesmer H., Bahramsoltani M., Schwerdtfeger K., Spangenberg L. (2021). Euthanasia distress and fearlessness about death in German veterinarians. Crisis.

[60] Tran L., Crane M.F., Phillips J.K. (2014). The distinct role of performing euthanasia on depression and suicide in veterinarians. J. Occup. Health Psychol..

[61] Witte T.K., Spitzer E.G., Edwards N., Fowler K.A., Nett R.J. (2019). Suicides and deaths of undetermined intent among veterinary professionals from 2003 through 2014. J. Am. Vet. Med. Assoc..

[62] Nett R.J., Witte T.K., Tomasi S.E., Fowler K.A. (2020). Storage of euthanasia solution as a factor in addressing veterinarian suicides. J. Am. Vet. Med. Assoc..

[63] Stamm B.H. The Concise ProQOL Manual, 2nd ed. https://proqol.org/.

[64] Matte A.R., Khosa D.K., Coe J.B., Meehan M., Niel L. (2020). Exploring pet owners’ experiences and self-reported satisfaction and grief following companion animal euthanasia. Vet. Rec..

